# A comparison of different GRACE solutions in terrestrial water storage trend estimation over Tibetan Plateau

**DOI:** 10.1038/s41598-018-38337-1

**Published:** 2019-02-11

**Authors:** Wenlong Jing, Pengyan Zhang, Xiaodan Zhao

**Affiliations:** 1 Guangzhou Institute of Geography, Guangzhou, 510070 China; 2 Key Laboratory of Guangdong for Utilization of Remote Sensing and Geographical Information System, Guangzhou, 510070 China; 3 Guangdong Open Laboratory of Geospatial Information Technology and Application, Guangzhou, 510070 China; 4 College of Environment and Planning, Henan University, Kaifeng, 475004 China; 5 Institute of Agriculture and rural Sustainable Development, Henan University, Kaifeng, 475004 China; 6 Collaborative Innovation Center on Yellow River Civilization of Henan Province, Henan University, Kaifeng, 475001 China

## Abstract

The Gravity Recovery and Climate Experiment (GRACE) terrestrial water storage anomalies (TWSA) estimations provide valuable information for the monitoring of land water resources changes. Multiple parameters and strategies for inversion of the water storage changes have been explored. The explorations on differences between GRACE solutions in local regions and basins are fundamental and important. This study focuses on comparisons of TWSA trends between different GRACE solutions over Tibetan Plateau (TP), both storage and flux among solutions were compared. Results show that great discrepancies exist in TWSA between GRACE solutions derived from the standard spherical harmonic approach (SSH) and the mascon approach. Three SSH-based GRACE solutions (JPL, CSR, and GFZ) detect no significant TWSA changes for the whole area of Tibetan Plateau, whereas JPL mascon solution (JPL-M) and CSR mascon solution (CSR-M) gave decreasing trends of 3.10 km^3^/yr and 3.77 km^3^/yr, respectively. This difference also exists in the Yangtze River-Yellow River basin (YYR basin) in the TP. Although five solutions derived consistent TWSA trends in northwest river basin (NWR basin) and southwest river basin (SWR basin) in the TP, the variations between different solutions are 2.88 km^3^/yr and 4.75 km^3^/yr for NWR and SWR basin respectively, which could not be neglected. The JPL-M solution, as a result, would overestimate both TWSA decreasing and increasing trends comparing with other GRACE solutions. The results of this study are expected to provide references for the studies of water resource dynamics over Tibetan Plateau and the surrounding areas based on GRACE TWSA products.

## Introduction

Monitoring water resources dynamics over Tibetan Plateau is extremely important for understanding the global water cycle and the regional responses to climate change over Tibetan Plateau and the surrounding areas^1–3^. It is helpful for water resources management and drought events detections over China and South Asian countries^4,5^.

The Gravity Recovery and Climate Experiment (GRACE) twin satellites, which were launched in 2002, have provided global monthly land or terrestrial water storage anomalies (TWSA) by measuring Earth’s gravity field changes^6^. The TWSA includes water storage anomalies of five components: snow water, canopy water, surface water, soil water, and groundwater^7^. The GRACE TWSA can reveal total water availability variations both storage and flux at continental scales^8,9^. Latest released versions of GRACE TWSA products include Spherical Harmonic Data Versions and Mascon Data Versions. The Spherical Harmonic Data Versions are processed by using a standard spherical harmonic approach^6^, and the Mascon Data Versions are produced by using mass concentration blocks (mascons)^10^. Three different processing centers, which are the GeoforschungsZentrum Potsdam (GFZ), the Center for Space Research at University of Texas, Austin (CSR), and the Jet Propulsion Laboratory (JPL), release different solutions by using different approaches and parameters.

These GRACE TWSA solutions, produced using different approaches by different centers, have been widely used for evaluation of water resources changes over large-scale regions and basins^11–16^. For TP, several researches have been conducted for the estimation of water mass balance based on the GRACE TWSA solutions. According to Jacob, T. *et al*.^17^, the TWSA over TP experienced an increasing at trend of 7.0 km^3^/yr from 2003 to 2010, which was obtained based on GRACE CSR mascon (CSR-M) solution. The TWSA trend over TP estimated from Guo, J. *et al*.^18^ based on GRACE CSR solution was 5.3 km^3^/yr during 2003 to 2012. However, the results from Pengkun, X. U. *et al*.^19^ indicated the TWSA decreased from 2005 to 2010 at a trend of −3.64 km^3^/yr, which the conclusion was drawn from estimations based on released version 4 of CSR solution (CSR-RL04). As can be seen that the TWSA trends estimated from different GRACE solutions revealed different water resource variations over TP. Such discrepancies between different GRACE solutions can also be found over some other basins around the world^7^. Differences between different GRACE solutions can lead to great discrepancies in TWSA trend estimations. Therefore, quantifying those discrepancies is significant and urgent for understanding uncertainties in monitoring water resources variations based on GRACE outputs at both continental and basin scales.

The objective of this study is to explore differences between GRACE solutions over Tibetan Plateau and evaluate the uncertainty arises from the choice of different GRACE solutions. Both storage and fluxes among GRACE solutions were compared. In addition, spatial pattern of GRACE TWSA trends and the variations among different GRACE solutions were also analyzed. The results of this study are expected to provide direct reference for the researches involving water resources dynamics over TP and the surrounding areas by using GRACE-derived products.

### Study area and data resources

#### Study area

**Figure 1:**
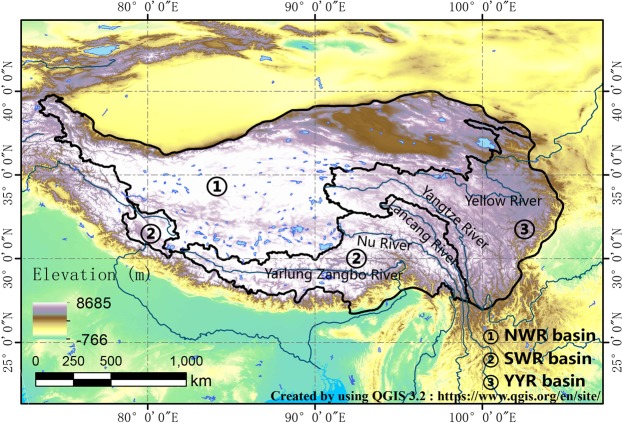
Spatial pattern of terrain and basin boundary of Tibetan Plateau (created by using QGIS 3.2: https://www.qgis.org/en/site/).

The Tibetan Plateau (TP) is the highest plateau on the earth, which is located between 26°00′N and 39°47′N, and 73°19′E and 104°47′E. The TP stretches from the southern margin of the Himalayas to the north, to the Kunlun Mountains, the Altun Mountains, and the northern margin of the Qilian Mountains. In the west, the Pamirs and Karakoram Ranges, and the east and northeast connect with the western section of the Qinling Mountains and the Loess Plateau^20,21^ (Fig. 1). The map in Fig. 1 was created by using the mapping tool in an open source software QGIS 3.2 (https://www.qgis.org/en/site/). The average altitude of TP is over 4000 meters above sea level^22^, thus it is commonly referred to as the “Roof of the World”^20^. The TP is the headstream of many rivers in East Asia, Southeast Asia and South Asia^23^. A number of world-class rivers flowing down from the Tibetan Plateau to the southeast, flowing into the sea, such as the Yarlung Zangbo River, Lancang River, and Nu River. The Yangtze River and the Yellow River, which are known as the mother rivers of Chinese people, also originate from east Tibetan Plateau^24^.

Continental rivers are mainly located in the northwest part of TP. The Yarlung Zangbo River, Lancang River, Nu River, which flow to the Indian Ocean, are in the southwest TP. The Yangtze River and Yellow River originate from east TP and flow down to the Pacific Ocean. Therefore, the TP is divided into three basins: the northwest river basin (NWR basin), the southwest river basin (SWR basin), and the Yangtze River and Yellow River basin (YYR basin). The area of each basin is 1336460 km^2^ (NWR basin), 628921 km^2^ (SWR basin), and 658504 km^2^ (YYR basin) (Fig. 1).

### GRACE data

#### The RL05 spherical harmonics

The Gravity Recovery and Climate Experiment (GRACE) twin satellites were launched in 2002. GRACE satellites provide global monthly total water storage anomalies by measuring Earth’s gravity field changes. The spherical harmonic approach (SSH) is the standard for the first decade of GRACE observations^6,25^. The official GRACE Science Data System continuously released monthly GRACE solution for three different processing centers: GeoforschungsZentrum Potsdam (GFZ), Center for Space Research at University of Texas, Austin (CSR), and Jet Propulsion Laboratory (JPL). These three solutions used different parameters and strategies, such as different degree and order, spherical harmonic coefficient, spatial filter and smoothing factor^26^. The current solutions are the Release-05 gravity field solutions (RL05). The three GRACE terrestrial water storage (TWS) solutions (JPL, CSR, and GFZ) were downloaded from https://grace.jpl.nasa.gov/data/get-data/monthly-mass-grids-land/. The original data were provided at 1 degree in both latitude and longitude and resampled at 0.5 degrees.

#### The Global Mascons

Mass Concentration blocks (mascons) are another form of gravity field basis function. The “mascon” make the implementing of geophysical constraints much easier and is a much more rigorous approach comparing with the standard spherical harmonic approach of empirical post-processing filtering^27,28^. The JPL mascon (JPL-M) and CSR mascon (CSR-M) solutions were provided at 0.5 degrees at https://grace.jpl.nasa.gov/data/get-data/monthly-mass-grids-land/. In this study, we compared the GRACE TWSA solutions during 2003 to 2016.

## Methods

### Time series decomposition and TWSA trend estimations

The original released GRACE TWSA outputs reveal the monthly variations of total water storage; therefore, seasonality should be firstly removed from the original TWSA series in order to estimate TWSA trends^29^. In addition, non-available data are filled with a simple temporal interpolation using the months either side^30^. Specifically, for one month of data gap, the missing value were interpolated by averaging the values of the two months either side; for period with more than one month of data gaps, a linear regression function was fitted by using the values of the months either side, then the missing values were estimated by applying the function to the gap months.

The harmonic analysis is commonly implemented approach for removing seasonal variations from time series data^10^. The Seasonal Trend decomposition using Loess (STL), according to Scanlon, B. R. *et al*.^7^, showed similar decomposed outputs with harmonic analysis. The STL, proposed by Cleveland *et al*.^31^ was reported to be a versatile and robust method for decomposing time series^32^. In this study, we used STL to decompose TWSA monthly time series. Key to the STL approach is LOESS (LOcal regrESSion) smoothing. LOESS fits a smoothed series *X(j*) to an input time series *X(j)* = *X(t*_*j*_*)*, where *t*_*j*_ is a sequence of discrete sampling times. The smoothed value at each point *j* is given by the value at time *t*_*j*_ of a polynomial fitted to the sampled values of *X* over a window (*j−q, j* + *q*), with decreasing weight assigned to points in this window as their distance from point *j* increases. The STL consists of outer and inner loops with a sequence of smoothing operator LOESS and generates three components from a time series^33^:

1 $${S}_{total}={S}_{long-term}+{S}_{seasonal}+{S}_{residual}$$

The long-term signal is then detrended into linear and non-linear trends by fitting a least squares linear regression and a cubic spline regression, respectively. The residuals reflect sub-seasonal signal and noise. Details of STL decomposition approach can be found in related publications^31,32^. The TWSA trends, as a result, refer to the linear trends estimated from the long-term (deseasonalized) TWSA after STL analysis.Moreover, we also implemented the Mann Kendall Trend Test (M-K test) to identify trends from deseasonalized TWSA series. M-K test, proposed by Mann 1945^34^ and furtherly studied by Kendall 1955^35^, is a nonparametric and robust test for identifying a trend in a time series. It has been widely used to detect trends in hydrologic data^36^. Details about the M-K test can be found in related references^37,38^.

### Uncertainty of GRACE data and TWSA trend

In this study, we estimated the variability of GRACE TWSA trends in two aspects:

Variability among different GRACE solutions and the variability among linear trends estimated from different GRACE solutions. The standard deviation of the five solutions and the corresponding linear trends were calculated, respectively. Then, the two standard deviations were combined by calculating their root sum of squares (RSS):

2 $$Uncertainy=\sqrt{{\rm{STD}}(TWS{A}_{i})+{\rm{STD}}(TWSA\,trend{s}_{i})}$$

where ${{TWSA}}_{i}$ stands for TWSA from different solutions, and ${{TWSA\; trends}}_{i}$ is the TWSA trends estimated from long-term TWSA of different GRACE solutions, STD is short for standard deviation.

## Results and Discussion

### GRACE TWSA trends and uncertainties over Tibetan Plateau

**Figure 2:**
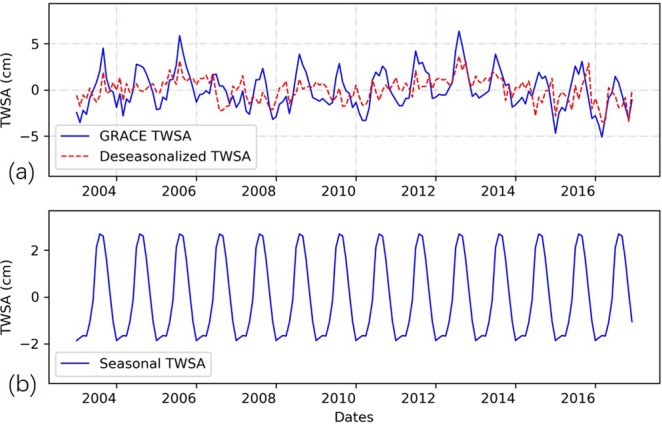
(**a**) The original TWSA signals and the deseasonalized TWSA of GRACE CSR solution in the TP, (**b**) the seasonal signals derived from GRACE CSR TWSA solution.

Long-term signal, seasonality, and residual of GRACE TWSA time series were decomposed by using the STL analysis. Figure 2(a) presents the original TWSA signals and the deseasonalized GRACE CSR TWSA in the TP. The seasonal signals were shown in Fig. 2(b). The seasonal variations, according to Fig. 2, were well removed from original GRACE TWSA time series. According the derived seasonal signals, the seasonal pattern of TWSA is similar to that of precipitation in TP region, lower in spring and winter and higher in summer and autumn. This is reasonable because summer precipitation dominates the annual precipitation in TP^39^, and abundant rainfall brings an increase in water reserves in summer. It is worth noting that TWSA peak in August, lagging one month than the peak month of precipitation of TP^39,40^. This is consistent with findings from previous researches that TWSA and precipitation show a one- or two-month lag^41,42^. One reason for the lagged response of TWSA to precipitation is the land itself would delay the response of TWSA to fluctuations in rainfall by delaying the infiltration. In addition, many other reasons would also contribute to the lagged response, such as runoff path length and vegetation density.

Then the TWSA trends were derived from the lone-term GRACE TWSA time series. Table 1 summarized linear trends and their variations of both TWSA storage and flux over Tibetan Plateau during the investigated period, and the M-K test results were also presented. According to the results, there are no significant TWSA trends derived from the solutions of the standard spherical harmonic approach (JPL, CSR, GFZ). The TWSA trends of JPL and GFZ are all 0.00, and the trends of TWSA storage and flux of CSR are -0.99 km^3^/yr and -0.04 cm/yr. However, the solutions produced by using global mascons (JPL-M and CSR-M) reveal decreasing trends over the TP. The TWSA storage trends of JPL-M and CSR-M are -3.10 km^3^/yr and -3.77 km^3^/yr, respectively. The variation of TWSA storage trend is 1.77 km^3^/yr.

**Table 1:** TWSA trends and the variations of different GRACE solutions over TP.

Solution	TWSA (km^3^/yr)	TWSA-flux (cm/yr)	M-K test trend	Variation (TWSA) (km^3^/yr)	Variation (TWSA-flux) (cm/yr)
JPL	0.00	0.00	no-trend	1.77	0.07
CSR	−0.99	−0.04	no-trend		
GFZ	0.00	0.00	no-trend		
JPL-M	−3.10	−0.12	decreasing		
CSR-M	−3.77	−0.14	decreasing		

**Figure 3:**
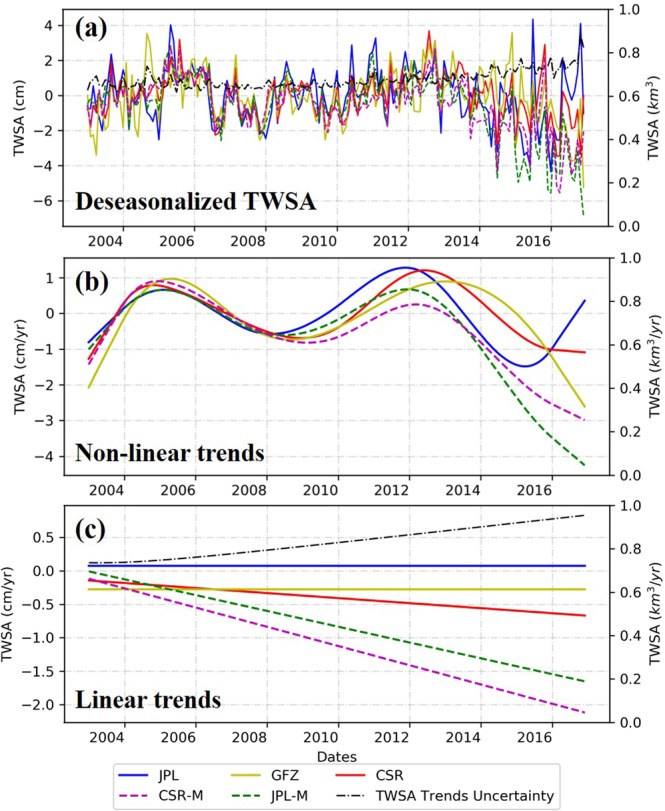
(**a**)Time series of deseasonalized GRACE TWSA of TP, (**b**) GRACE TWSA non-linear trends of TP, (**c**) GRACE TWSA linear trends of TP.

Figure 3 shows the time series of deseasonlized TWSA and the non-linear and linear trends. The variations of TWSA solutions are also displayed. In general, the TWSA over the TP experienced two increasing periods (2003 to 2004, 2008 to 2011) and two decreasing periods (2005 to 2008, 2013 to 2016). Variation (TWSA trends uncertainty) of TWSA arises from year of 2011, similar findings also can be found in some other basins, such as Brahmaputra, Brazos, Euphrates, Lena, Mississippi, Missouri, and Nile^7^. But there are also multiple basins that show different patterns of GRACE TWSA variations. Reasons leading to the differences are still unclear and remain investigation on a few relating factors, including: GRACE model structure, climate forcing, human intervention. The JPL-M and CSR-M solution monitored evident decreasing TWSA trends after 2012, and the slopes of linear trends of JPL and GFZ are close to zero. In general, the TWSA trends derived from five solutions show a similar pattern of fluctuations, but the variation between five solutions increases after 2011. The overall TWSA tendency derived from JPL, CSR, and GFZ is no-trend throughout the investigated period, whereas the solutions based on global mascons give decreasing trends of TWSA over Tibetan Plateau.

### GRACE TWSA trends and uncertainties in different basins

**Figure 4:**
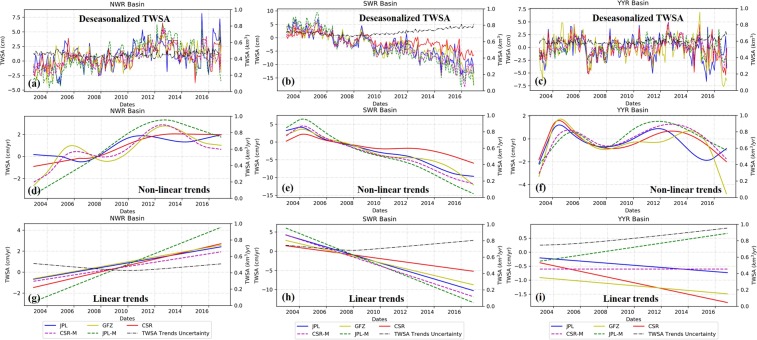
Time series of deseasonalized GRACE TWSA of (**a**) NWR Basin, (**b**) SWR Basin and (**c**) YYR basin, GRACE TWSA non-linear trends of (**d**) NWR Basin, (**e**) SWR Basin and (**f**) YYR basin, and GRACE TWSA linear trends of (**g**) NWR Basin, (**h**) SWR Basin and (**i**) YYR basin.

Figure 4 shows the temporal behaviors of different GRACE TWSA solutions for different river basins. Table 2 summarized TWSA trends for different solutions over the three basins of TP.

**Table 2:** TWSA trends and variations for different GRACE solutions over three basins of TP.

Basin	Solution	TWSA (km^3^/yr)	TWSA-flux (cm/yr)	Trend	Variation (TWSA) (km^3^/yr)	Variation (TWSA-flux) (cm/yr)
NWR	JPL	2.96	0.22	increasing	2.882	0.016
	CSR	4.01	0.30	increasing		
	GFZ	3.07	0.23	increasing		
	JPL-M	6.81	0.51	increasing		
	CSR-M	2.70	0.20	increasing		
SWR	JPL	−6.54	−1.04	decreasing	4.747	0.120
	CSR	−3.00	−0.48	decreasing		
	GFZ	−5.22	−0.83	decreasing		
	JPL-M	−8.75	−1.39	decreasing		
	CSR-M	−7.27	−1.16	decreasing		
YYR	JPL	−0.25	−0.04	no-trends	0.178	0.004
	CSR	−0.67	−0.10	no-trends		
	GFZ	−0.28	−0.04	no-trends		
	JPL-M	0.47	0.07	increasing		
	CSR-M	0.02	0.00	increasing		

The five solutions all reveal positive TWSA values over the NWR basin, with TWSA between 2.70 km^3^/yr and 6.81 km^3^/yr and TWSA flux ranging from 0.20 cm/yr to 0.51 cm/yr. The trends detected by using M-K test are also increasing for different solutions. According to Fig. 4, uncertainties of TWSA in NWR basin reveal stable temporal characteristics. The JPL-M solution shows the highest TWSA value among five solutions. This is also can be implied from time series of non-linear and linear trends in Fig. 4. The trend of JPL-M significantly deviates from other four solutions, and its temporal behavior also differs from other solution. In addition, the CSR-M and GFZ solutions display similar temporal trends according to the no-linear trends displayed in Fig. 4. In general, the JPL-M solution gives the highest TWSA comparing with other four solutions over NWR basin, and the CSR-M solution gives the lowest TWSA estimation. Variation between four solutions is 2.882 km^3^/yr, higher than the TWSA trends estimated by CSR-M solution, and marginally lower than that estimated by JPL solution.

The TWSA in the SWR basin decrease significantly during the entire period. The TWSA estimated from five solutions give consistent trends of decreasing, with TWSA trends between -8.75 km^3^/yr and -3.00 km^3^/yr. The decreasing trend of TWSA derived from JPL-M is 8.75 km^3^/yr, which is the highest among the TWSA trend estimations of the five solutions. The CSR TWSA gives the smallest decreasing trend estimations of 3.00 km^3^/yr, lower than the variations of the five trends (4.747 km^3^/yr). According to the temporal behaviors of deseasonalized TWSA and its trends displayed in Fig. 4, the uncertainties of TWSA and the trends increase after the year of 2009. The five solutions, in general, show similar time series characteristics during the entire period in SWR basin.

The variation between TWSA trends estimated from five solutions is 0.178 km^3^/yr in YYR basin, much smaller than that of other basins. This is because the TWSA trends detected is weak. Five solutions give different TWSA trends, ranging from -0.67 km^3^/yr to 0.47 km^3^/y. The TWSA trends of JPL, CSR, and GFZ are negative, and the JPL-M and CSR-M gives positive trends. The M-K test results of JPL, CSR, and GFZ TWSA trends reveal no-trend of TWSA, and the results of JPL-M and CSR-M are increasing. According to the time series, the uncertainties of TWSA trends arises after 2006. The JPL-M TWSA presents contrary trends to other four solutions.

### Spatial pattern of linear trend analysis and the differences

**Figure 5:**
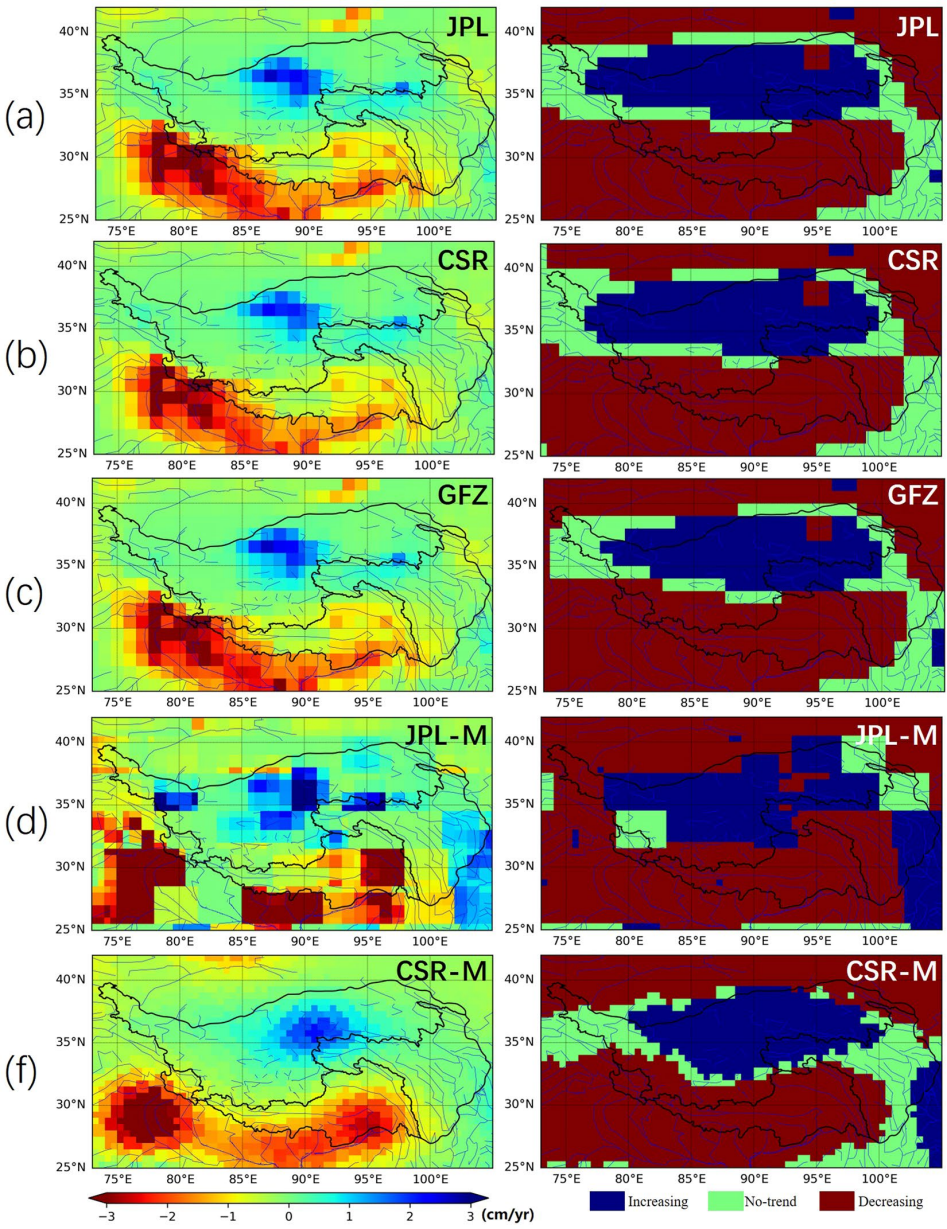
The spatial patterns of TWSA linear trends of different GRACE solutions (left) and the corresponding M-K test results (right): (**a**) JPL, (**b**) CSR, (**c**) GFZ, (**d**) JPL-M, and (**e**) CSR-M.

Fig. 5 shows the spatial patterns of TWSA linear trends of different GRACE solutions and the corresponding M-K test results. Five GRACE solutions all detect the TWSA increasing regions in the central part of NWR basin, which is Hoh Xil Nature Reserve. This is consistent with the conclusion of Xiang, L. *et al*.^43^. In the past decades, glaciers and snow-caps in the surrounding areas are experiencing shrinkage, leading to the increased runoff recharges in this area, and increasing precipitation in the eastern Pamir also can lead to the increasing water balance in the area^44^.

In addition, the Chinese Ecological Protection and Construction Project successfully helps in keeping the groundwater in Hoh Xil Nature Reserve and also the Three Rivers Source Region in the YYR basin^43^. However, spatial pattern and locations of the TWSA increasing regions between the spherical harmonic (JPL, CSR, GFZ) and mascons solutions (JPL-M, CSR-M) are different. The extent of increasing regions of CSR-M is larger than the other solutions. The high TWSA trend region of JPL-M solution is further east than those of other four solutions.

The SWR basin, which includes part of Himalayas and Yarlung Zangbo River, has experienced TWSA decreasing trends. Great TWSA decreasing trends can be found along the Himalayas and its surroundings. Significant TWSA decreasing trends were detected by the mass loss signals over east Himalayas area, which agrees well with some previous researches^18,43^. This is likely caused by the ice melting and decreased precipitation in the Himalayas^44^, but this conclusion need to be drawn carefully after further intensive studies.

**Figure 6:**
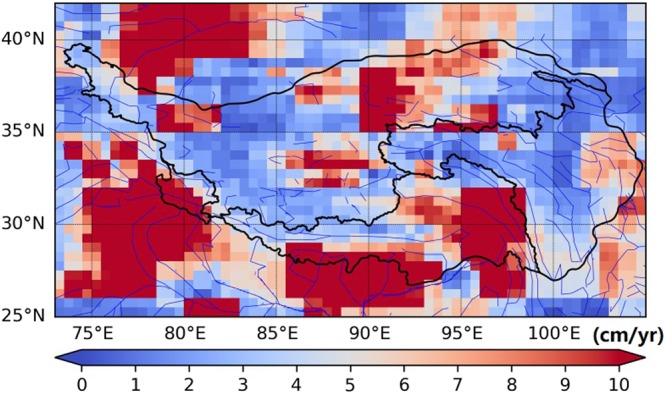
Spatial pattern of the standard deviation between TWSA-flux trends derived from five GRACE solutions.

Figure 6 shows the spatial distribution of standard deviation of TWSA-flux trends derived from five GRACE solutions. Variations between different solutions is notable over central part of NWR basin and east part of SWR basin. In the south Himalayas and west Himalayas, variations were also conspicuous. The significant differences, by comparing the spatial pattern of TWSA-flux trends in Fig. 6, are mainly caused by the disagreement area between solutions from the standard spherical harmonic approach and the global mascons approach. The TWSA trends generated solutions derived based on the standard spherical harmonic approach show similar spatial pattern, but the spatial pattern of TWSA trends deriving from two mascons solutions are much different.

The spherical harmonics have been well studied and many parameter choices and solution strategies have been explored by JPL, CSR, and GFZ. Spatial differences between the centers have been reported small over most parts of the world^26^. The analysis results in this study also support the findings. However, the spherical harmonic solutions suffered from poor observability of east-west gradients, which could lead to so-called stripes. Although the stripes can be removed by using smoothing methods, some real geophysical signals may be removed during the smoothing^27^.

**Figure 7:**
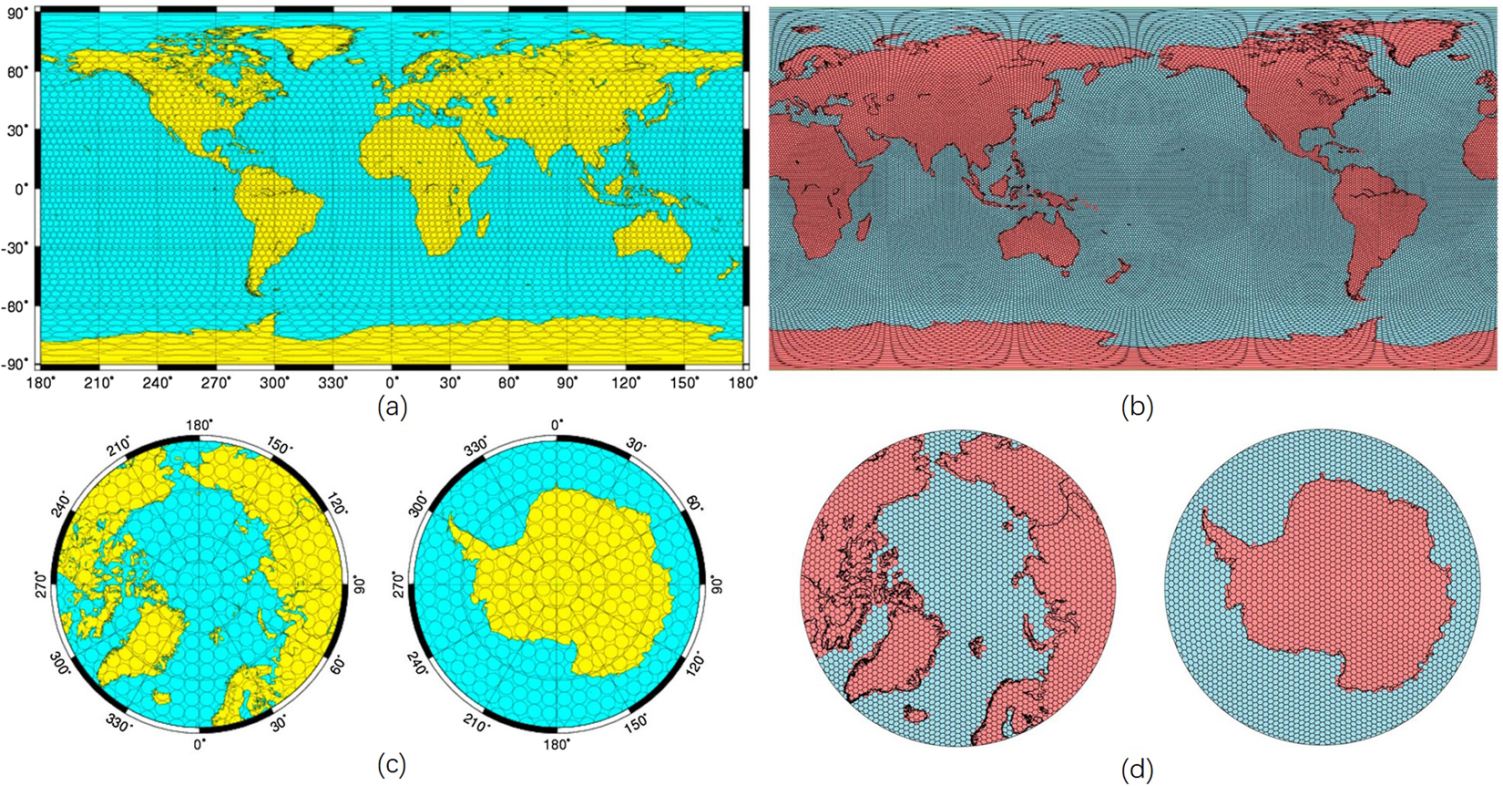
(**a**) Global 3° equal-area spherical cap mascons used for JPL-M solution, (**b**) global view of hexagonal tiles used for CSR-M solution, (**c**) global 3° equal-area spherical cap mascons in North Pole view (left) and South Pole view (right) for JPL-M solution, (**d**) North Pole view (left) and South Pole view (right) of hexagonal tiles for CSR-M solution^27,45^.

On theory, the mascon approaches surpass the spherical harmonics solutions because it relies less on empirical scale factors to obtain accurate mass estimations. The two mascon solutions considered in this study were both developed based on global mass concentration blocks, however, the JPL-M used global 3° equal-area spherical cap mascons, and the CSR-M used a collection of hexagonal tiles with about 1° equatorial longitudinal distance^10,27^ (Fig. 7). As is can be seen from Fig. 7, the spherical cap solution used by JPL-M cannot cover the entire Earth’s surface, as gaps exit between each spherical cap. This discrepancies between CSR-M and JPL-M solutions is observable through the visual comparison from Fig. 5 that the signals in the CSR-M solution (Fig. 5(e)) shows more spatial details and have gradual change along adjacent 1° mascons as compared to the sharp step change seen between adjacent 3° mascons in the JPL-M solution (Fig. 5(d)). As a result, the CSR-M solution used in this study is preferred due to its more user-friendly estimation algorithm and higher apparent resolution. However, this study only evaluated discrepancies in between GRACE TWSA solutions in Tibetan Plateau, future research should explore differences between GRACE solutions in different regions.

## Conclusion

Comparison of five GRACE TWSA solutions (JPL, CSR, GFZ, JPL-M, and CSR-M) over Tibetan Plateau in this study reveals that significant differences can be found between SSH-based solutions (JPL, CSR, GFZ) and mascon-based solutions:

1. For the whole Tibetan Plateau, the changes in TWSA obtained by the five solutions were slightly different, and no significant changes were detected by the JPL, CSR, and GFZ. But the results from the JPL-M and CSR-M showed that the TP’s TWSA showed an overall decreasing trend of 3.10 km^3^/yr and 3.77 km^3^/yr, respectively. For TWSA time series, five solutions showed similar fluctuation patterns, but after 2011, the differences were large, and the uncertainty increased.
2. Five solutions produced consistent TWSA trends in NWR and SWR basin, but the TWSA trend values vary among different solutions. For the NWR basin, five solutions all produced increasing trends, with the TWSA trends between 2.70 km^3^/yr and 6.81 km^3^/yr, and the JPL-M solution estimated the highest TWSA trends among five GRACE solutions. TWSA in SWR basin show increasing trends, with TWSA trends between -8.75 km^3^/yr and -3.00 km^3^/yr, and the JPL-M trends also produced the highest TWSA decreasing trends. In YYR basin, the solutions from the SSH approach disagreed with the two solutions from the mascons. The JPL, CSR, GFZ solution did not detect significant trends in YYR basin, whereas JPL-M and CSR-M solution reveals increasing trends.
3. TWSA in central part of NWR basin significantly increased during 2003 to 2016. This is probably caused by the runoff from surrounding glaciers/snow-caps and increasing precipitation, and the Chinese Ecological Protection and Construction Project may also have helped to keep the water resources in Hoh Xil Nature Reserve and also the Three Rivers Source Region. Large decreasing can be detected along Himalayas and its surroundings, which is likely owing to the ice melting in the Himalayas according to some previous studies.
4. Standard deviations of TWSA trends between five solutions for three basins are 2.882 km^3^/yr (NWR), 4.747 km^3^/yr (SWR), and 0.178 km^3^/yr (YYR). Spatial patterns of TWSA trends of three SSH-based solutions are consistent, and large variations can be found in the mascons-based solutions. Significant differences exist in the central part of NWR basin, east SWR basin, and south and west Himalayas. In addition, the JPL-M generally overestimate both increasing and decreasing trends of TWSA comparing with other four solutions.

This study investigated the GRACE-derived TWSA trends over Tibetan Plateau and identified the uncertainties of TWSA and trends among five GRACE solutions. The results of this study provide guidelines for investigations in terrestrial water storage changes over Tibetan Plateau and its surrounding areas based on GRACE derived TWSA datasets. Analysis of differences among GRACE solutions in other local basins would be furtherly studied in the future work to identify the uncertainties of GRACE solutions in regional water balance researches.

## Data Availability

The datasets generated and analyzed during the current study are available from the first and corresponding author on reasonable request for research purpose.
